# A New Way to Assess the Trajectory of Remission in Crohn Disease: Correlating Drug Levels With a Noninvasive Blood Biomarker of Mucosal Remission

**DOI:** 10.1093/crocol/otab036

**Published:** 2021-06-12

**Authors:** Anas Gremida, Parakkal Deepak

**Affiliations:** Division of Gastroenterology, Washington University in St. Louis School of Medicine, St. Louis, Missouri, USA

Over the years, there has been an evolution in our understanding of the treatment goals and targets in patients with Crohn disease (CD). The goal of treatment has evolved from clinical remission to target a deeper level of remission at the mucosal level with transmural healing being an adjunct target.^1^ This evolving treat-to-target concept encompasses optimizing the therapeutic benefit of the various mechanisms of actions available to treat CD, followed by an iterative process utilizing noninvasive biomarkers and invasive endoscopic procedures to achieve and maintain remission defined by the chosen target, most appropriately endoscopic healing.

There are multiple factors that may determine the success of achieving the goal of endoscopic healing through an impact on the pharmacokinetics of the specific mechanisms of action. While many factors determining the exposure–response relationship are nonmodifiable such as gender, age, body mass index, tumor necrosis factor (TNF) level, disease phenotype, and history of prior treatment failure; increase in trough concentrations of the drug can be achieved through therapeutic drug monitoring (TDM) by a combination of dose escalation and prevention of antidrug antibody formation using a concomitant immunomodulator.^2,3^ The main goal of TDM in inflammatory bowel disease is to optimize drug levels and maximize the chances of remission.

Assessment of the treatment target of mucosal healing is typically achieved via endoscopy. However, endoscopy is an invasive costly procedure, has the burden of bowel preparation and it may not always be able to assess the target if disease is restricted to the small bowel especially in the presence of a stricture at the ileocecal valve or ileocolonic anastomosis or if CD is intramural or skipping the terminal ileum.^4,5^ Additionally, most clinicians do not use currently available endoscopic scoring systems in routine clinical practice, which makes the objectivity of endoscopic assessment and comparison between serial procedures during longitudinal follow-up challenging.

Until now, both fecal calprotectin and serum C-reactive protein (CRP) were the only available noninvasive inflammatory biomarkers that could be routinely ordered in clinical practice to assess the trajectory of remission. Their role as biomarkers to guide a treat-to-target approach were highlighted in the phase 3 CALM study (Efficacy and Safety of Two Treatment Algorithms in Adults With Moderate to Severe Crohn’s Disease).^6^ In the control arm, adjustments of therapy were based solely on clinical symptoms. In the treat-to-target arm, changes were based on both clinical symptoms, as well as levels of CRP and fecal calprotectin. The study found that patients in the treat-to-target arm had superior outcomes compared to the control group. In a post hoc analyses, fecal calprotectin was the main marker that drove the decision to escalate therapy compared to CRP (62% vs 46% at week 11; 56% vs 46% at week 23). This reflects the limitations of current blood-based biomarkers in accurately reflecting mucosal disease activity. Additional limitations of fecal calprotectin include day–day variations depending on the timing of stool collection, CD phenotype, and disease location as well as other reasons for false-positive and false-negative values of calprotectin. Moreover, patients have expressed a preference for blood-based biomarkers over stool-based ones due to the difficulty in obtaining and depositing stool samples making fecal calprotectin a less desirable test.^7^ Therefore, there has been a unmet need for a new blood-based test to assess mucosal healing.

A newly launched validated serum-based assay known as “Monitr test” provides more comprehensive assessment of disease activity in patients with CD through measurement of 13 different inflammatory and matrix remodeling factors. These markers are CRP, CEACAM, VCAM, TGF-alpha, interleukin-7, amyloid A, angiopoietin-1 and -2, matrix metalloproteases (MMP1, MMP2, MMP3, MMP9), and extracellular matrix metalloproteinase inducer (EMMPRIN).^8^ These markers reflect the degree of inflammatory burden in the mucosa and can be used longitudinally to assess response to treatment. The value from these biomarkers is combined in an algorithm the endoscopic healing index (EHI) score, which ranges from 0 to 100 where 0 indicates no or mild disease with 92% probability and 100 represents an active disease with 82% of probability. EHI score of 50 has a slightly lower probability of active disease at 78%. A serum cutoff value of <20 was found to be associated with endoscopic remission with a high sensitivity and specificity and suggested as a cutoff to indicate endoscopic remission in clinical practice.

In this issue of CC360, in a large retrospective study, samples from over 1700 CD patients, including both adults and children, were analyzed to study the relationship between the drug levels of infliximab (IFX) and adalimumab (ADA) and EHI, and to determine the targeted drug level of IFX and ADA that is associated with EHI <20.^9^ The study concluded that a linear inverse relationship existed between the levels of IFX and ADA and EHI with a median anti-TNF concentration associated with EHI <20 of 11.1 µg/mL for IFX and 9.2 µg/mL for ADA. These levels are higher than the previously recommended threshold for IFX (≥5 µg/mL) and ADA (≥7.5 µg/mL) which were based on clinical disease activity rather than endoscopic activity.^10^ This study has thus raised the ceiling of therapeutic levels associated with mucosal remission.

The main limitations of this study are that the data were analyzed from a retrospective laboratory database which makes it hard to control for multiple cofounding factors that may impact mucosal healing including the underlying disease phenotype and location, whether the samples were draw at trough and clinician-specific factors that went into dose escalation and type of TDM (reactive vs proactive). It also lacked data for correlation with endoscopic disease activity and clinical outcomes. Even though mucosal healing is the most widely accepted definition of deep remission, CD is a transmural disease and even in patients with sustained mucosal healing, residual bowel wall inflammation can still be detected by cross-sectional imaging.^11^ There are also now a few studies that suggest the superiority of transmural healing over mucosal healing.^12,13^ In a substudy of the TAILORIX cohort that analyzed magnetic resonance enterography data using MaRIA scoring system at baseline and week 54 in CD patients, there was correlation between the endoscopic and radiologic disease activity at baseline but a disconnect at week 54.^14^ It also showed that transmural radiologic remission correlated with IFX trough level at week 14 when the IFX trough level cutoff value at 7.8 µg/mL. This level is much lower than the median IFX levels needed to achieve an EHI <20. This then raises the question whether EHI is reliable noninvasive tool to reflect both mucosal and transmural healing or whether the levels reflect the nontrough nature of some of the samples.

The findings of this study require prospective validation in other cohorts with IFX and ADA drug levels, endoscopic disease activity and further correlation with transmural healing using radiographic disease activity data. Once validated, it may become an additional tool in our armamentarium to achieve and then monitor a remission target in a patient with CD, reducing the need for repeat endoscopy and potentially cross-sectional imaging.
